# Validation of functional polymorphisms affecting maize plant height by unoccupied aerial systems discovers novel temporal phenotypes

**DOI:** 10.1093/g3journal/jkab075

**Published:** 2021-04-05

**Authors:** Alper Adak, Clarissa Conrad, Yuanyuan Chen, Scott C Wilde, Seth C Murray, Steven L Anderson II, Nithya K Subramanian

**Affiliations:** 1 Department of Soil and Crop Sciences, Texas A&M University, College Station, TX 77843, USA; 2 National Key Laboratory of Crop Genetic Improvement, Huazhong Agricultural University, Wuhan 430070, China; 3 Department of Environmental Horticulture, Institute of Food and Agricultural Sciences, Mid-Florida Research and Education Center, University of Florida, Apopka, FL 32703, USA

**Keywords:** unoccupied aerial system, high-throughput phenotyping, temporal loci effects

## Abstract

Plant height (PHT) in maize (*Zea mays* L.) has been scrutinized genetically and phenotypically due to relationship with other agronomically valuable traits (*e.g.*, yield). Heritable variation of PHT is determined by many discovered quantitative trait loci; however, phenotypic effects of such loci often lack validation across environments and genetic backgrounds, especially in the hybrid state grown by farmers rather than the inbred state more often used by geneticists. A previous genome-wide association study using a topcrossed hybrid diversity panel identified two novel quantitative trait variants controlling both PHT and grain yield. Here, heterogeneous inbred families demonstrated that these two loci, characterized by two single nucleotide polymorphisms (SNPs), cause phenotypic variation in inbred lines, but that size of these effects were variable across four different genetic backgrounds, ranging from 1 to 10 cm. Weekly unoccupied aerial system flights demonstrated the two SNPs had larger effects, varying from 10 to 25 cm, in early growth while effects decreased toward the end of the season. These results show that allelic effect sizes of economically valuable loci are both dynamic in temporal growth and dynamic across genetic backgrounds, resulting in informative phenotypic variability overlooked following traditional phenotyping methods. Public genotyping data show recent favorable allele selection in elite temperate germplasm with little change across tropical backgrounds. As these loci remain rarer in tropical germplasm, with effects most visible early in growth, they are useful for breeding and selection to expand the genetic basis of maize.

## Introduction

Plant height (PHT) in maize has been subjected to many phenomic and genomic investigations since it influences plant architecture and agricultural performance, relating to other agronomically and economically significant traits in maize (*Zea mays* L.) (Sari-gorla *et al.* 1999; Sibov *et al.* 2003; Lima *et al.* 2006; Farfan *et al.* 2013, 2015; Peiffer *et al.* 2014; Anderson *et al.* 2019). A key component of success to the green revolution was the manipulation of PHT in wheat (*Triticum spp.*) and rice (*Oryza Sativa*) through the introduction of dwarf loci, initially used as a breeding strategy to maintain grain yield lost through lodging (Khush 2001; Peng *et al.* 1999). However, an important postscript has been that taller PHT leads to better yields in a number of cereal crops including rice (Zhang *et al.* 2017), sorghum (Murray *et al.* 2008; Shukla *et al.* 2017), wheat (Navabi *et al.* 2006), and maize (Farfan *et al.* 2013); as long as lodging can be avoided. Specifically, Farfan *et al.* (2013) found that manual measured terminal PHT was positively correlated (*r* = 0.61) with grain yield in commercial hybrids over subtropical environments. They proposed that an optimal taller PHT is a desirable maize ideotype with respect to yield, especially under subtropical heat and drought stress, as long as lodging is not an issue.

The wealth of studies on maize PHT has demonstrated the complexity, dynamic pattern, and polygenic inheritance of this trait; a trait governed by a large number of loci but with minor effects (Peiffer *et al.* 2014; Wallace *et al.* 2016; Wang *et al.* 2019). Thus far at least 219 quantitative trait loci (QTLs) have been identified as controlling the PHT in maize (http://archive.gramene.org/qtl/). Very few of these to our knowledge have been confirmed as QTL in independent studies across different genetic backgrounds and environments.

In contrast, the large effect genes identified with maize PHT have been associated with novel mutant alleles in hormone pathway genes; alleles rare or absent in landrace and elite cultivars because they are deleterious to plant fitness in nature. For instance, the dwarfing genes *dwarf 8* and *dwarf 9* encode DELLA proteins, which repress gibberellin (GA)-induced gene transcriptions in the absence of GA signaling (Lawit *et al.* 2010); the *Dwarf3* gene (*D3*) of maize has significant sequence similarity to the cytochrome P450, which encodes one of the early steps in GA biosynthesis (Winkler and Helentjaris 1995); *brachytic2* mutants, the polar movement of auxins was hindered, which resulted in compact lower stalk internodes (Multani *et al.* 2003), and *nana plant1* effects brassinosteroid synthesis (Hartwig *et al.* 2011).

That quantitative genetic loci discovered for PHT diversity still segregating in maize have not been cloned, let alone manipulated has likely been due to (i) limitations in detection ability of height related QTLs in diverse structure of mapping populations (Xu *et al.* 2017), (ii) different growth pattern under different plant architectures and genetic backgrounds (El-soda *et al.* 2014; Pigliucci 2005), (iii) reaction norms across varying environments and genetic-by-environmental interactions (El-soda *et al.* 2014; Gage *et al.* 2017), and (iv) antagonistic pleiotropy of major genes (Peiffer *et al.* 2014). This is likely compounded by the use of inbred lines in genetic mapping as opposed to testcrossed hybrids. Maize evolved as a heterogenous and heterozygous outcrossing species and inbred lines expose weakly deleterious alleles uncommonly exposed in nature which are detected but which heterosis in hybrids can again mask (Yang *et al.* 2017). Hybrids tend to reduce phenotypic variance, especially when topcrossed to a common tester.

A genome wide association study (GWAS) on testcrossed hybrids made between a diversity panel and topcrossed to a line from the Stiff Stalk heterotic group (Tx714; Betrán et al. 2004) under variable management discovered three significant loci associated with both terminal PHT and yield (Farfan *et al.* 2015). These loci explained up to 5.6 cm per variant (4.6% of total), two of which (Chr2: 27,482,431kp and Chr7: 164,955,163 kp; maize refgen_v2) also ranged from 0.14 ton/ha to 0.59 ton/ha effects on grain yield (4.9% of total). While Farfan *et al.* (2015) suggest possible candidate genes, they did not calculate the linkage disequilibrium (LD) from these single nucleotide polymorphisms (SNPs) or exhaustively examine linked candidates, which we do here in this article. The two candidate genes suggested by Farfan *et al.* (2015) include GRMZM2G035688 and GRMZM2G009320. GRMZM2G035688 is an important crop improvement gene in maize that is responsible for arrangement the maize leaves around stem (referring the aberrant phyllotaxy (*abph1*) in maize) (Hufford *et al.* 2012; Jackson and Hake 1999). GRMZM2G009320, a housekeeping gene and acts as a glycose-related enzyme, encodes the glyceraldehyde-3-phosphate dehydrogenase (GAPDH) enzyme to regulate the energy metabolism in maize (Bustos *et al.* 2008; Zhang *et al.* 2011). Even if the metabolic and developmental-related functions of these genes have been identified, the temporal effect sizes of native alleles on phenotype across maize development stages and under different genetic populations remain unknown.

Past GWASs have shown false positives due to cryptic population structure, familial relatedness, allele variants with low frequency or various allelic variants, as well as spurious associations between phenotypic variations and unlinked markers. For this reason, loci must be validated using different populations, environments (Larsson *et al.* 2013), and, where relevant, growth stages. Next to transformation or gene editing, near isogenic lines (NILs) remain the standard for the validation of effect sizes of loci on phenotype, crucial for plant breeders and geneticists to measure effect sizes of these loci.

Outside of Farfan *et al.* (2015), hybrid maize populations have been used in relatively few other GWASs to discover SNPs. GWAS can comprise both additive and nonadditive SNP effects for the traits controlled by both overdominance and dominance conditions (Warburton *et al.* 2015; Wang *et al.* 2017; Vidotti *et al.* 2019; Galli *et al.* 2020). So that validation of SNPs discovered in maize hybrid GWAS populations over multiple genetic backgrounds is important to find pure additive effects of candidate genes. Chen (2016) found effects consistent with Farfan *et al.* (2015) in constructed recombinant inbred line (RIL) populations as both inbred and hybrids; however, due to various field issues, this study did not have enough power to determine significance. RILs were thus used as the basis for developing the heterogenous inbred families (HIFs), a type of NIL, tested in this study.

In this study for the first time (i) validated the temporal loci effects, first discovered using hybrid genetic background in GWAS, in HIFs generated from different parental crosses; (ii) implemented a unoccupied aerial system (UAS) platform to detect temporal changing of these loci effects on PHTs of HIFs; (iii) examined epistasis between these two loci; and (iv) characterized genetic architecture of their pleotropic effects on flowering times

## Materials and methods

### Development of HIF populations

The two target SNPs were first validated to segregate across elite breeding lines by means of Sanger sequencing, as expected from the genotyping calls in the previous GWAS (Farfan *et al.* 2015). These calls were further confirmed using F_1_ hybrids on-hand that were derived from these parents (Chen 2016). The primers for Sanger sequencing were developed by Primer 3 (Untergasser *et al.* 2012), using the B73 maize genome (Schnable *et al.* 2009) as reference; the primer information is provided in Supplementary Table S1. All polymorphisms within the linkage populations were identified using ClustalX 2.1 (Larkin *et al.* 2007). As a result, LH82, LAMA, Tx740, Ki3, and NC356 were used as parental lines in four linkage populations (Chen 2016) and HIFs since their genotyping calls were validated to segregate (Supplementary Figure S1).

**Figure 1:**
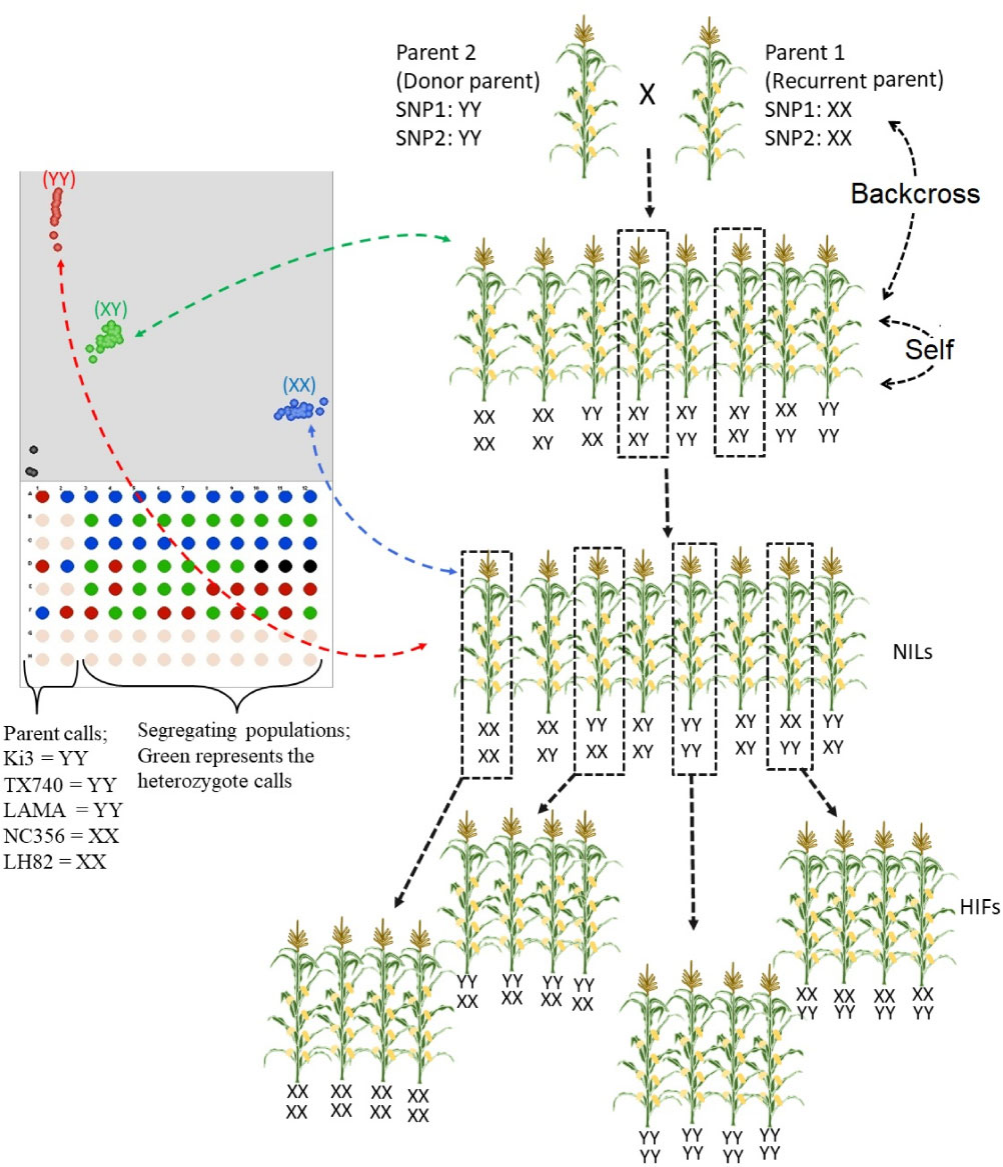
Breeding scheme of generating HIFs based on two SNP models and selection stages of pedigrees via KASP technology (http://www.kbioscience.co.uk/). Ten to 20 plants from each plot were randomly selected or aided by markers for multiple generations until obtaining NILs (BC_3_F_2_ or more recurrent parent crosses or selfs). Only those having heterozygous loci (XY) were selected each generation and their ears were grown as rows (ear-to-row selection). After obtaining NILs, homozygous calls from both SNPs were selected as both identical (XX:XX, YY:YY) and opposite (XX:YY, YY:XX) to generate HIFs. All parents were genotyped (left). Parents; Ki3, NC356, Tx740, and LH82, calls (SNP1: SNP2) are YY:YY, XX:XX, YY:YY, and XX:XX, respectively. No template controls, black color in KASP figure, were used in each plate as negative controls.

The four linkage populations, segregating for the two SNPs of interest, were developed from crosses (1) LH82 × LAMA, (2) Ki3 × NC356, (3) NC356 × Ki3, and (4) Tx740 × NC356 (recurrent parent × donor parent for populations 1 to 4), respectively, and selfed to generate F_5_ RILs (Chen 2016). RILs were selected based on having the desired donor SNPs on a mostly recurrent parent background and backcrossed to the recurrent parent to create F_1_ hybrids. First, F_1_ hybrids were further backcrossed with recurrent parents (four to five times) and selfed (three to five times) up to obtaining NILs as HIFs. Until obtaining NILs, both loci (SNP1: 27,482,431 kp in Chr2; SNP2: 164,955,163 kp in Chr7 based on Maize Refgen_v2) were maintained as heterozygote calls in each population (seen as X: Y; *i.e.*, donor allele: recurrent parent allele in Kompetitive Allele-Specific PCR (KASP) genotyping results (below and Figure 1). Second, individuals were selected in each population to have both opposite (XX: YY and/or YY: XX) and identical (XX: XX and/or YY: YY) to determine the HIFs within each population (Figure 1).

### DNA extraction and KASP genotyping of HIFs

Total genomic DNA was extracted from the frozen (−60°C) plant flag leaf tissue using a modified cetyltrimethylammonium bromide method (Chen and Ronald 1999). To design the unique markers targeting the SNP1 and SNP2, around 100 bp surrounding the two SNPs on either side were selected to determine allele-specific primers and allele general SNPs using BatchPrimer3 v1.0 (You *et al.* 2008). Sequence information of primers was obtained from Chen (2016). Loci implemented into KASP (http://www.kbioscience.co.uk/) assays by Chen (2016) were used in marker-assisted backcrossing to develop HIFs across different NIL backgrounds and used to detect SNP calls (XX, XY, and YY) for developing HIFs during 2016 to 2019 (Figure 1).

### Determining LD

Farfan *et al.* (2015) did not provide LD estimates, so the data were reanalyzed and are reported here. Tassel software (version 5) (Bradbury *et al.* 2007) was used to obtain LD (LD windows size = 10 markers). First, LD decay plots were generated per chromosome using 61.402 total polymorphic markers used in Farfan *et al.* (2015) to determine the LD decay rate. Especially, LD decay plots were generated to assess distances of LD decay pattern in chromosomes 2 and 7 where SNP1 and SNP2 were discovered. (Supplementary Figure S9). Second, nearby LD patterns of SNP1 (Chr2: 27,482,431 kp) and SNP2 (Chr7: 164,955,163 kp) were visualized using LD heatmap in R (Shin *et al.* 2006). LD calculated based on *R*^2^ and lower than 0.2 LD was ignored. The MaizeGBD (http://www.maizegdb.org/) genome browser was used to determine plausible genes linked to SNPs. The Gramene database (http://www.gramene.org) was used for the identification of candidate genes.

### Allele frequency estimates in elite germplasm

The Panzea (https://www.panzea.org/) website was used to extract sequence information of genes from publicly available maize germplasm to evaluate how the allele frequency of these SNPs differs over germplasm and time. For elite temperate material, the information on the years when germplasm was developed were obtained from expired plant variety protection (Ex-PVP) certificates available on the USDA-ARS Germplasm Resources Information Network (https://www.ars-grin.gov). Allele shift of the loci were illustrated as count-based frequency histogram (qualitative calculation) through release years of germplasms.

### Planting and agronomic practices

Plants were grown near College Station, TX (coordinate: 30°33′00.8″N 96°26′04.3″W) for summer nurseries and Weslaco (26°09′32.7″N 97°57′36.1″W), Texas for winter nurseries from 2016 to 2019. All nurseries were grown based on range and row design with two replications per HIF. Each row plot (∼6.10 m long) in each range contained two row plots of two different HIFs. Plot rows were 3.05 m long for each HIF, and 18 seed were planted per HIF row plots. During the advancement of HIFs from 2016 to 2018, SNP1 and SNP2 calls were primarily maintained by selecting heterozygotes (X:Y) to advance and increase. For traditional and UAS phenotyping in College Station 2019, entire plots of X:X, Y:Y, and X:Y for each HIF were planted on the 12th of April, 2019, in two replicates. These HIFs were grown in a total of 18 ranges with 16 row plots each as well as parental lines and red stalker inbreds (Supplementary Table S3). Row plots of red stalker inbreds were used as planting indicators to verify that the planting was correct via orthomosaic because of their red stem and leaf color. Experimental designs were applied as a split:split:split plot design where the main split was replicate, the second split was population/genetic background, and the third split was genotype. Unless noted, all reported hand measurements and unoccupied aerial vehicle (UAV) flights were conducted when HIFs were grown near College Station in 2019.

### Phenotyping

Days to anthesis (DTA) and silking (DTS) were recorded on a plot basis when 50% of the plants were showing anthers and silks, respectively, checking plots daily. Three different terminal PHT measurements were taken using a ruler including TH, FH, EH July 2nd, 2019, about 2 to 3 weeks after flowering. In addition, UAV (aka drone) PHT measurements were taken weekly from emergence to the end of the growth period. The flight dates were shown as day/month/year (dd/mm/yy). Grain yield was not taken as it has little value in the inbred lines screened which often are confounded by inconsistent pollination in the heat stress of Texas.

UAV images of the field were taken using a DJI Phantom 4 Pro V2.0 (DJI, Shenzhen, China) at an above ground altitude of 25 m. The standard integrated camera resulted in images having a resolution of 72 DPI. DJI standard flight control software was used. Orthomosaics and point clouds were created with the images for each flight by using Agisoft Metashape V15.2 software (Agisoft LLC, Russia). The captured images were at 72 dpi with 90% overlap and were used to create an orthomosaic and point cloud for determining the PHT as a function of time during the growth period. Ground control points were used during the flights to assist the data processing and reduce effects due to aberrations and the resulting georeferenced mosaics.

Previous work has shown that various methods to measure inbred maize plants from the ground using point clouds produced similar results (Anderson *et al.* 2020). Point clouds of each flight were processed using CloudCompare (version: 2.11. alpha). To set a canopy height model (CHM), first flight containing bare ground was used as a digital terrain model (DTM). Digital surface model (DSM) of each flight was subtracted from DTM to calculate CHM (Supplementary Figure S2). Each plot was drawn using the polygon function of CloudCompare.

### Statistical inference

Statistical models were developed according to the distribution of SNP1 and SNP2 combinations obtained from the HIFs. Spatial variation was partitioned as random effects into ranges and rows. Each model was run using a restricted maximum likelihood method in JMP version 15.0.0 (SAS Institute Inc., Cary, NC, USA) to predict the best linear unbiased estimates (BLUEs) of SNPs. SNPs were fit as fixed effects to obtain BLUEs values for flights as well as for ruler measurements. Separate models with genotypes as random effects in an all random model were fit to obtain variance components. All components, except the SNPs and population, were always fit as random effects under the following mixed linear models in each model.

First, each SNP was tested separately within each population (Equation 1). While one of two SNPs was segregating, the other one was fixed (not segregating as XX or YY) in respective populations to compare the BLUEs of SNP calls. This equation was used for hand measurement data on a plant basis for each population.

Equation 1 $$Y_{\mathrm{ijkl}}=\mu +{\mathrm{SNP}}_{i}+{\mathrm{Range}}_{j}+{\mathrm{Row}}_{k}+{\mathrm{Rep}}_{l}+\varepsilon_{\mathrm{ijkl}}$$

Within this base model, response variable $\left(Y_{\mathrm{ijkl}}\right)$ was one of the three hand measures of PHT data; (${\mathrm{SNP}}_{i})$ represented variance of one of SNPs to be tested on condition that other one is fixed XX and/or YY within each respective population. Other variance components, including range $\left({\mathrm{Range}}_{j}∼N(0,\;\sigma_{\mathrm{Range}}^{2}\right)$, row $\left({\mathrm{Row}}_{k}∼N(0,\;\sigma_{\mathrm{Row}}^{2}\right)$, and rep $\left({\mathrm{Rep}}_{l}∼N(0,\;\sigma_{\mathrm{Rep}}^{2}\right)$, account for the spatial variation. $\varepsilon_{\mathrm{ijkl}}∼N\left(0,\;\sigma^{2}\right)\;$is the pooled unexplained residual error.

PHT and flowering time were also tested for SNP1 and SNP2 individually combining all data across populations 1, 2, and 3 (Equation 2). While one of the two SNPs segregated, the other one was fixed (not segregating as XX) in the model. In this equation, the population (${\mathrm{Pop}}_{i})$ effect was added compared to Equation 1. BLUEs and BLUPs of SNPs and their interactions with populations, respectively, were obtained for each UAS flight and ruler measurement.

Equation 2 $$Y_{\mathrm{ijklm}}=\mu +{\mathrm{Pop}}_{i}+\;{\mathrm{SNP}}_{j}+{\left[\mathrm{Pop}*\mathrm{SNP}\right]}_{ij}+{\mathrm{Range}}_{k}+{\mathrm{Row}}_{l}+{\mathrm{Rep}}_{m}+\epsilon_{\mathrm{ijklm}}$$

The interactions of both SNPs and populations using the full factorial function were tested for both flowering time and for PHT from the ruler measurement and UAS flights temporally across populations 1 and 2 (Equation 3).

Equation 3 $$Y_{\mathrm{ijklmn}}=\mu +{\mathrm{Pop}}_{i}+\;{\mathrm{SNP}1}_{j}+{\mathrm{SNP}2}_{k}+{\left[\mathrm{Pop}*\mathrm{SNP}1\right]}_{ij}+{\left[\mathrm{Pop}*\mathrm{SNP}2\right]}_{ik}+{\left[\mathrm{SNP}1*\mathrm{SNP}2\right]}_{jk}+{\left[\mathrm{Pop}*\mathrm{SNP}1*\mathrm{SNP}2\right]}_{\mathrm{ijk}}+{\mathrm{Range}}_{l}+{\mathrm{Row}}_{m}+{\mathrm{Rep}}_{n}+\epsilon_{\mathrm{ijklmn}}$$

Here, response variable $\left(Y_{\mathrm{ijklmn}}\right)$ is PHT data. ${\mathrm{SNP}1}_{j}$, ${\mathrm{SNP}2}_{k}$, and ${\mathrm{Pop}}_{i}∼N(0,\;\sigma_{\mathrm{Pop}}^{2})$ represent the variance components of SNP1, SNP2, and population, respectively, while other variance components were the same as stated previously in Equation 1 and Equation 2. In this equation, only populations 1 and 2 were used due to sample size.

Orthogonal contrasts were applied to ${\mathrm{SNP}}_{i}$ and ${\left[\mathrm{Pop}*\mathrm{SNP}\right]}_{ij}$ variance components in Equation 2 as well as ${\left[\mathrm{SNP}1*\mathrm{SNP}2\right]}_{jk}$ and ${\left[\mathrm{Pop}*\mathrm{SNP}1*\mathrm{SNP}2\right]}_{\mathrm{ijk}}$ in Equation 3 to illustrate temporal statistically significance differences between BLUEs of loci calls. In Equation 2, BLUEs of XX and YY calls of two SNPs were orthogonally contrasted for each SNP and each population, while BLUEs of XX:XX (SNP1:SNP2) and other call combinations (XX:YY, YY:XX, and YY:YY) were contrasted for SNP1 and SNP2 interactions as well as SNPs and population interactions in Equation 3. Statistically significance differences between calls for each time point were reported at the level of 0.01, 0.05, and 0.001 in Figures 3–6.

Repeatability (R) was calculated based on following formula with number of replication (*r*) for single environments (Equation 4).

Equation 4 $$\mathrm{Repeatability}\;(R)=\frac{\sigma_{\mathrm{Pop}}^{2}}{\sigma_{\mathrm{Pop}}^{2}+\;\sigma_{\epsilon }^{2}/r}$$

Additional data processing and visualizations were performed in R version 3.5.1 (R Core Team 2018).

### Data availability

Unoccupied aerial vehicle (UAV)-point cloud data (.laz files), processing reports (.pdf files), tif files, belonging to 05/17/19, 05/30/19, 06/04/19, 06/11/19, 06/13/19 (mm/dd/yy) flight dates, are available at https://doi.org/10.6084/m9.figshare.13046306.v4. Ruler-based plant height measurements (Ruler measurement.xlsx), canopy height measurements derived from UAV-point cloud data (Uav-chm.xlsx), Field map (Field Map.xlsx), and Experimental area (Experimental area .pdf) are available at https://doi.org/10.6084/m9.figshare.13046306.v4. UAV-point cloud data (.laz files), processing reports (.pdf files), tif files, belonging to 06/19/19, 06/21/19, 06/28/19, 07/02/19, 07/09/19 and 07/12/19 flight dates, are available at https://doi.org/10.6084/m9.figshare.13269953.v1.

Supplementary material is available at https://doi.org/10.25387/g3.14188481.

Primer development and designs used in KASP genotyping are given in Supplementary Tables S1 and S2. Table S3 contains the number of row plots of HIFs with their population background and SNPs information. Supplementary Tables S4 and S5 contain the results of explained percent variations estimated by Equations 2 and 3, respectively, for ruler measurements. Supplementary Figure S1 GWAS Manhattan plots, LD of SNPs, allelic effects, and parental sequences of previous work are confirmed by this study. Previously, two SNPs were discovered for plant height as well as for yield using the plant height as a covariate in a GWAS (Farfan *et al.* 2015). (a) Physical position of the two SNPs on Manhattan plot when plant height was included as a covariate in the model to predict yield. Zoom in figures of two SNPs on chromosomes 2 and 7 and lengths of the genes in kilobase pairs (Kb). (b) SNPs positions updated from maize-NAM reference genome version 5 were used to find LD using *R*^2^ values and flanking regions of the genes for the two SNPs. (c) Effects sizes for the two SNPs (tonne per hectare). (d) Polymorphic SNPs colocalized in LD blocks and haplotype variants based on two SNPs and (e) segregations of two SNPs in parental genotypes, advanced populations used in this study as follows: [LAMA (recurrent parent) × LH82], [Ki3 × NC356 (recurrent parent)], [Ki3 (recurrent parent) × NC356] and [Tx740 (recurrent parents) × NC356]. Supplementary Figure S2 Illustrations of canopy height measurements (CHM) obtained by extracting the digital surface model (DSM) from DTM. The orthomosaic obtained from the drone flight that was flown on June 28, 2019, is shown as an example in here. C2C (cloud to cloud) absolute distances (as meters unit) heatmap show the plant heights of HIFs in the point clouds of CHM after the extraction of point clouds of DSM from point clouds of DTM. Viridis color heatmap was used to illustrate the plant heights in the ranges and row plots as top view. The zoomed row plot illustrates the side view example of plant height differences between two heterogeneous inbred families developed from same population background comparatively; one of those has both favorable alleles (XX:XX; SNP1:SNP2), the other has unfavorable alleles (YY:YY; SNP1:SNP2). Supplementary Figures S3 and S4 contain the BLUEs for SNPs and the interaction of SNPs with populations obtained by Equation 2 for ruler measurements. Supplementary Figure S5 contains the BLUEs for flowering times estimated by Equation 3. Supplementary Figures S6 and S7 contain the BLUEs for the interactions between both SNPs and combined interactions between SNPs and populations, respectively, for ruler measurements estimated by Equation 3. Supplementary Figure S8 contains Pearson correlations between UAS-PHT with ruler measured means and median. Supplementary Figure S9 contains the LD decay plots for each chromosome.

## Results

**Figure 2:**
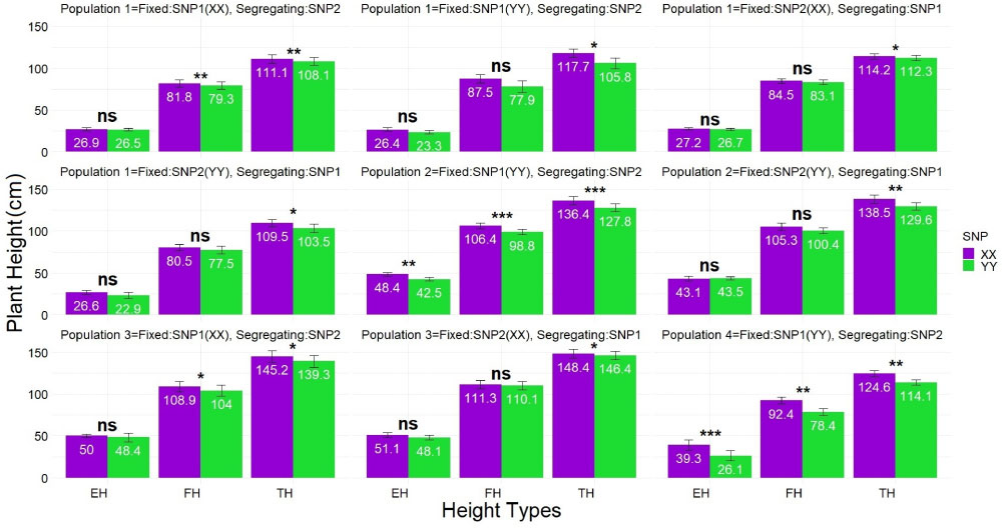
BLUEs of all three ruler measures of plant heights. This showed XX calls significantly increased all height measures in a consistent direction across populations. Population 1, 2, 3, and 4 are NILs of [LAMA (recurrent parent) × LH82], [Ki3 × NC356 (recurrent parent)], [Ki3 (recurrent parent) × NC356], and [Tx740 (recurrent parents) × NC356], respectively. BLUEs were calculated using Equation 1$\left({\mathrm{SNP}}_{i}\;\mathrm{term}\right)$. Differences of BLUEs between XX and YY calls were statistically significant across all populations for TH which changed between 2.0 cm and 8.9 cm for SNP1 and between 3.0 cm and 11.9 cm for SNP2. *, **, and *** indicate significance levels at 0.05, 0.01 and 0.001, respectively, while ns indicates not significant. Whiskers represent the standard error. TH, tip of tassel height; FH, flag leaf collar height; and EH, height of the first ear shank from ground on the x-axis.

The effects of cytosine/C for SNP1, adenine/A for SNP2 (*e.g.*, XX) calls in both SNPs, contributed by both NC356 and LH82 parents (Supplementary Figure S1), increased all three ruler measures of PHTs (TH; from ground to tip of tassel, FH; from the ground to the flag leaf collar, EH; first ear height from the ground to first ear shank). Tassel height differences between XX and YY calls were statistically significant across all populations (Figure 2), varying from 2.0 cm to 8.9 cm (SNP1) and 3.0 cm to 11.9 cm (SNP2) depending on the populations genetic background (Figure 2). The favorable locus (XX) of SNP1 and SNP2 across populations increased TH ∼ 4 cm and FH ∼ 3 cm (Equation 2; Supplementary Figure S3). Interactions between SNP1*population and SNP2*population varied, with TH differences were observed up to 10 cm, followed by up to 7.0 cm for FH (Supplementary Figure S4). Flowering times (DTA and DTS) when used as response in Equation 2 demonstrated that the taller XX allele of SNP1 and SNP2 for PHTs also caused later flowering. XX allele of SNPs delayed flowering times between 1 day and 5 days depending on the genetic backgrounds of populations (Supplementary Figure S5). Result of orthogonal contrasts conducted between calls of each population showed this lateness was statistically significant (Supplementary Figure S5).

In Equation 3, SNP1 and SNP2 interactions ${\left[\mathrm{SNP}1*\mathrm{SNP}2\right]}_{jk}$ for TH and combined interaction with populations ${\left[\mathrm{Pop}*\mathrm{SNP}1*\mathrm{SNP}2\right]}_{\mathrm{ijk}}$ were found to be significantly taller than shortest combination (YY-YY) when either SNP1, SNP2, or both were XX favorable locus, resulting in that combined favorable SNP1 and SNP2 loci (XX-XX) was tallest in TH, which was 8.8 cm taller than the YY-YY combination (Supplementary Figure S6). This was 3.5 cm taller than expected from SNP1 or SNP2 alone and represents a synergistic effect between these two loci. There was also an epistatic effect of these loci with the XX-XX combination increasing height 8 cm in population 1 but 9.6 cm for population 2 which was consistent for other measurements of PHT (Supplementary Figure S7).

The proportion of total experimental variance attributable to differences between populations ($\sigma_{\mathrm{Pop}}^{2}$) varied from 64% to 80% within Equation 2 and Equation 3 for PHT measurements by ruler. Population effects, spatial (range, row) partitioned large amounts of experimental variance, but repeatability was high at 89% to 95% (Supplementary Tables S4 and S5).

### Statistical inferences of UAS PHT

**Figure 3:**
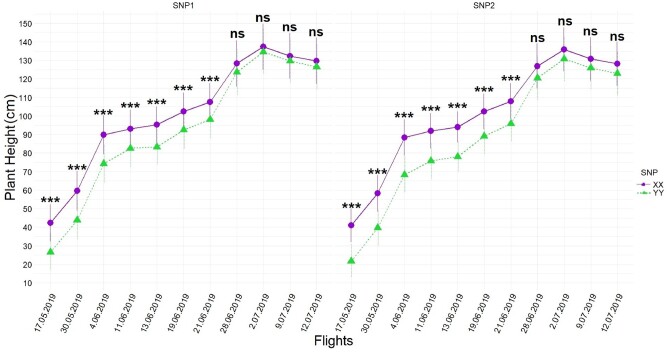
Temporal resolution of differences between SNP1 (left) and SNP2 (right) calls obtained by Equation 2$\left({\mathrm{SNP}}_{j}\;\mathrm{term}\right)$ during UAS flights across all populations. Whiskers represent the standard error. BLUEs of calls (XX *vs* YY) were orthogonally contrasted for each SNP at each time point and statistically significant differences were placed above the effects. *** indicates significance level at 0.001, while ns indicates not significant.

**Figure 4:**
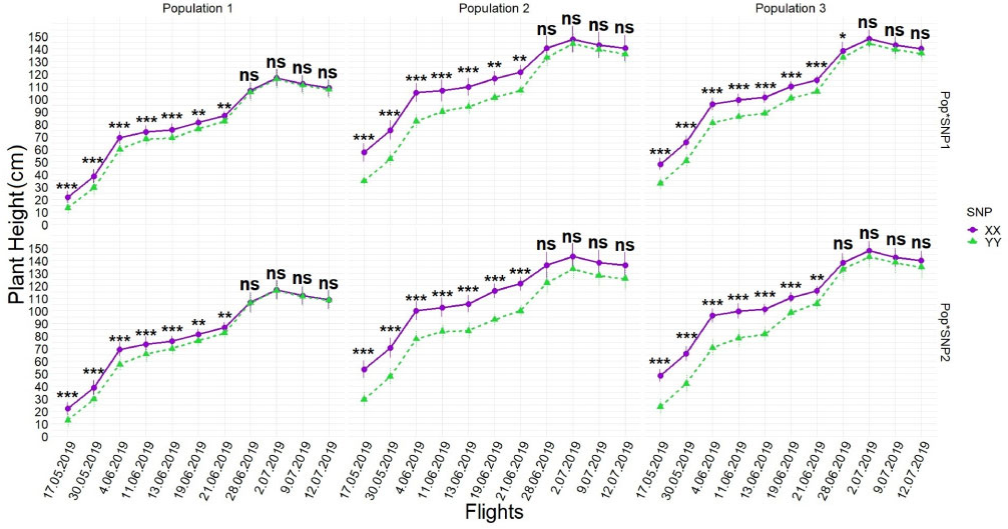
Temporal resolution of interactions of ${\left[\mathrm{Pop}*\mathrm{SNP}\right]}_{ij}$ obtained by Equation 2 during UAS flights. Modeling interactions showed that there were large differences between how the SNPs behaved on different genetic backgrounds. Whiskers represent the standard error. BLUEs of calls (XX *vs* YY) were orthogonally contrasted for each SNP in each population at each time point and statistically significant differences were placed above the effects for each time points. *, **, and *** indicate significance levels at 0.05, 0.01, and 0.001 respectively, while ns indicates not significant.

Temporal resolution of each UAS flight captured that the highest PHT (Canopy Height Model; CHM) differences between favorable (XX) and unfavorable loci (YY) were 16–20 cm in early growing stages (34–54 days after sowing; first four flights) but narrowed 3–5 cm by harvest time depending on when either SNP1 or SNP2 was tested in Equation 2, respectively (Figure 3). The differences between favorable and unfavorable loci varied depending on the interaction between populations with SNP1 ${\left[\mathrm{Pop}*\mathrm{SNP}1\right]}_{ij}$ and populations with SNP2 ${\left[\mathrm{Pop}*\mathrm{SNP}2\right]}_{ik}$ by Equation 2. The differences between calls in either interaction had a descending pattern from early growing season to time of harvest, showing the highest differences between calls for populations were captured between 9 cm and 26 cm in early season and narrowed 1 cm to 10 cm by the time of harvest (Figure 4).

**Figure 5:**
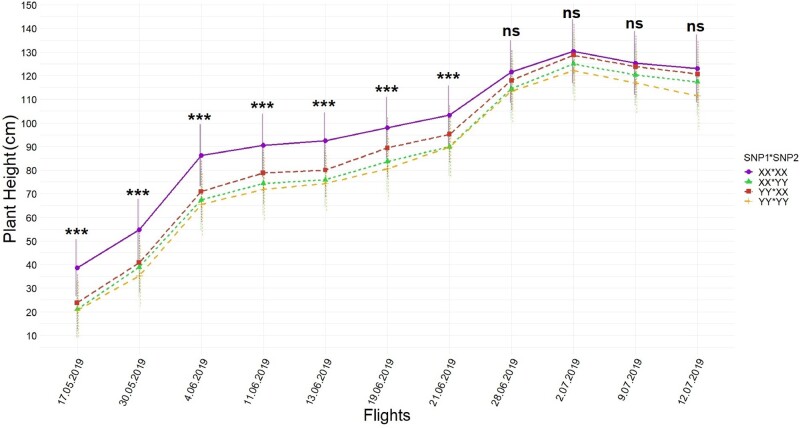
Temporal resolution of differences among SNP1-SNP2 interactions during UAS flights. The interaction$\;{\left[\mathrm{SNP}1*\mathrm{SNP}2\right]}_{jk}$was obtained from Equation 3 and shows that the two loci had a synergistic effect on increasing height. Whiskers represent the standard error. BLUEs of XX:XX (SNP1:SNP2) and other call combinations (XX:YY, YY:XX, and YY:YY) were contrasted for SNP1 and SNP2 interactions at each time point and statistically significant differences were placed above the effects for each time points. and *** indicate significance levels at 0.05, 0.01, and 0.001 respectively, while ns indicates not significant.

**Figure 6:**
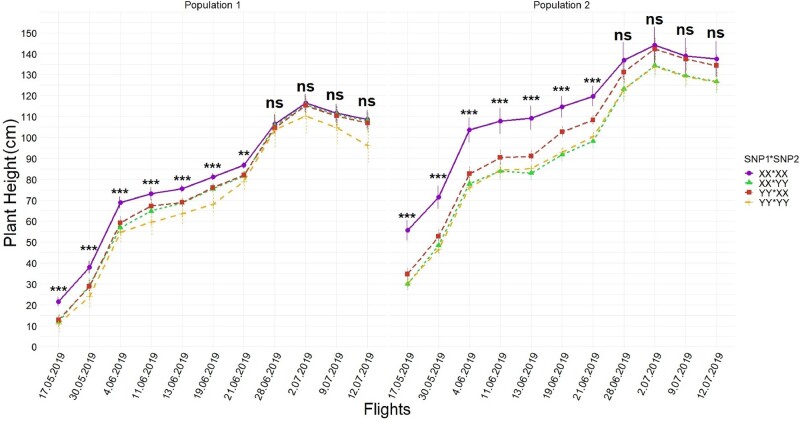
Temporal resolution of differences for two populations among SNP1-SNP2 interactions during UAS flights. Interactions ${\left[\mathrm{Pop}*\mathrm{SNP}1*\mathrm{SNP}2\right]}_{\mathrm{ijk}}$ obtained from Equation 3 showed the SNP combinations had different effects across different populations genetic backgrounds, especially early in the season. Whiskers represent the standard error. BLUEs of XX:XX (SNP1:SNP2) and other call combinations (XX:YY, YY:XX, and YY:YY) were contrasted for SNPs and population interactions at each time point and statistically significant differences were placed above the effects for each time points. and *** indicate significance levels at 0.05, 0.01, and 0.001 respectively, while ns indicates not significant.

In Equation 3, UAS captured that favorable loci combinations of XX-XX (SNP1: SNP2) were tallest in every flight followed by YY-XX, XX-YY, and YY-YY (Figure 5), resulting in height differences between favorable and unfavorable loci combined for population 1 and population 2 of 11–25 cm in the early growing stages and 7–10 cm by the time of harvest (Figure 6). Synergetic effects of the favorable loci combination on the unfavorable loci combination also decreased from 9 cm to 2 cm as the growing period progressed.

Population variation ($\sigma_{\mathrm{Pop}}^{2}$) always explained the highest percentage of total variation in both Equation 2 and Equation 3, resulting in repeatability estimates which fluctuated between 84% and 97% (Tables 1 and 2) during growing periods for PHT. SNP1 ${(\sigma }_{\mathrm{SNP}1}^{2})$ and SNP2 ${(\sigma }_{\mathrm{SNP}2}^{2})\;$in Equation 2 showed decreasing trends from ∼20% to 30% of explained total variation to below 1% over the growing period (Table 1) as well as decreases from ∼2% to 5% to below 1% in the interaction of SNPs in Equation 3 (Table 2)

**Table 1:** Percentages of total variance explained by each component in Equation 2 when SNP1 was tested (above) and SNP2 was tested (below) as well as the total variance in number and repeatability for each UAS flight^a^*, **, and *** indicate significance levels at 0.05, 0.01, and 0.001 respectively.

Variance component (Random effect)	Percentage of variation explained by each variable component for each flight										
	17.05.19	30.05.19	4.06.19	11.06.19	13.06.19	19.06.19	21.06.19	28.06.19	2.07.19	9.07.19	12.07.19
Population	45.7	46.2	45.5	47.1	47.2	64.3	66.0	54.0	53.8	54.3	54.1
SNP1	20.4	18.1	18.9	9.1	13.9	8.1	7.6	0.7	0.3	0.3	0.4
Population*SNP1	2.6	1.8	1.7	1.7	1.8	1.1	1.3	0.0	0.0	0.0	0.0
Replication	8.0	9.4	8.5	7.4	7.8	4.9	4.7	14.7	14.5	14.2	14.0
Row	0.2	0.3	0.0	1.1	0.5	0.5	0.6	3.4	3.2	3.3	3.2
Range	11.7**	13.0**	13.8**	14.3**	15.9**	12.4***	10.9**	7.0*	7.5*	7.2*	7.7*
Residual	11.4	10.8	11.7	19.3	12.9	8.7	9.0	20.3	20.7	20.7	20.6
Total variation in number	449.4	490.1	476.7	474.9	412.5	395.4	371.8	547.8	551.3	550.1	559.3
Repeatability (R)	0.89	0.89	0.87	0.83	0.88	0.94	0.94	0.84	0.84	0.84	0.84
Population	30.9	32.3	32.8	34.7	32.2	50.9	88.2***	48.4***	50.5***	49.2***	82.0***
SNP2	32.4	27.6	30.8	21.9	24.2	16.6	0.1***	0.1***	0.2***	0.1***	0.1***
Population*SNP2	7.1	5.8	3.9	4.3	7.9	7.1	0.0	0.1***	0.1***	0.1***	0.1***
Replication	9.2	11.3	9.3	12.2	7.8	6.2	0.4	30.4	27.3	28.0	7.2
Row	0.1	0.1	0.1	0.1	0.2	0.8	0.7	0.8***	0.6***	0.4***	1.0***
Range	11.9**	14.3**	14.8**	17.2**	16.3**	11.8**	5.7***	7.4***	7.3***	7.3***	2.7***
Residual	8.4	8.6	8.2	9.7	11.3	6.6	4.9	12.9	14.0	14.9	6.8
Total variation in number	475.2	512.6	548.9	473.8	394.7	403.0	385.2	660.4	484.1	608.2	1379.2
Repeatability (R)	0.88	0.88	0.89	0.88	0.85	0.94	0.97	0.88	0.88	0.87	0.96

a The flight dates were shown as day/month/year.

**Table 2:** Percentages of variance explained by each component in Equation 3 as well as total variance and repeatability for each UAS flights^a^ . *, **, and *** indicate significance levels at 0.05, 0.01, and 0.001respectively.

Variance component (Random effect)	Percentage of variation explained by each variable component for each flight										
	17.05.19	30.05.19	4.06.19	11.06.19	13.06.19	19.06.19	21.06.19	28.06.19	2.07.19	9.07.19	12.07.19
Population	81.4***	81.1***	74.6***	81.1***	79.3***	68.3	84.8***	70.8***	57.4***	57.3***	57.4***
SNP1	2.2***	2.7***	2.1***	1.5***	1.6***	1.4	0.1***	0.6***	0.7***	0.6***	0.3***
Population*SNP1	0.1***	0.1***	0.1***	0.1***	0.1***	0.1	0.1***	0.3***	0.5***	0.6***	0.6***
SNP2	5.5***	3.9	5.7	3.4	3.2	4.1	0.3***	2.0***	1.2***	1.0***	0.2***
Population*SNP2	0.1***	0.2***	0.2***	0.1***	1.6***	6.1	3.5***	1.4***	2.2***	2.6***	2.2***
SNP1*SNP2	0.7***	0.7***	1.2***	1.1***	1.8***	2.0	1.7***	0.2***	0.3***	0.3***	0.7***
Population*SNP1*SNP2	0.7***	0.2***	0.1	0.1***	1.1***	0.1***	0.1***	0.1***	0.1***	0.1***	0.1***
Replication	0.7	1.4	1.3	1.3	0.0	0.0	0.0	7.4	5.1	4.8***	5.2
Row	0.2***	0.1***	0.1	0.1***	0.5***	1.1	0.9***	2.3***	2.9***	2.8***	2.8***
Range	3.3***	5.0***	7.2**	5.2***	3.7***	6.4*	2.4***	4.2***	11.2***	11.2***	11.5***
Residual	5.1	4.5	7.4	6.0	7.1	10.3	6.1	10.7	18.3	18.7***	19.1
Total variation in number	640.1	807.7	609.1	895.6	691.2	377.0	593.0	871.2	473.8	463.0	466.0
Repeatability (R)	0.97	0.97	0.95	0.96	0.95	0.93	0.96	0.93	0.86	0.86	0.86

a The flight dates were shown as day/month/year (dd/mm/yy).

### Accuracy assessment between UAS-PHT and TH

For accuracy assessment, means and medians of each plot measured by ruler on July 2nd, 2019, were correlated with UAS-PHT captured on the same date, and a correlation coefficient was found to be 0.83 for either the median or mean correlated with UAS-PHT (Supplementary Figure S8).

### Candidate genes associated with the SNPs

LD decay distances calculated for each chromosome were found to be 1.5, 5.8, 4.5, 3.7, 4.5, 5.1, 4.5, 4.5, 4.9, and 5.7 kb for chromosomes 1 to 10, respectively (Supplementary Figure S9). Candidate genes were determined based on the LD decay around the surrounding regions of SNP1 (Chr2: 27,482,431 kb) and SNP2 (Chr7: 164,955,163 kb) as well as their physical positions using the Maize Refgen v2 coordinates (Supplementary Figure S2). SNP1 (Chr2: 27,482,431 kb) has a strong LD ($R^{2}$:1, sig = 0.00) with an adjacent locus (Chr2: 27,482,479 kb) which is 48 base pair away (upstream region) and both loci are in the genic region of GRMZM2G035688 (Chr2: from 27,478,703 to 27,483,682 kb) genes (Supplementary Figure S2). The region 5.8 kb upstream and downstream of SNP1 was also investigated, since the LD decay distance chromosome 2 was 5.8 kb. Only one other gene, GRMZM2G035637 (Chr2: from 27,478,035 to 27,479,631 kb), falls within the downstream region of SNP1 (1 kb away).

SNP2 (Chr7: 164,955,163 kb) has strong LD ($R^{2}$:0.86, sig = 0.95) with a locus (Chr7: 164,954,968 bp) that is located at 195 bp away downstream region of SNP2. SNP2 and the locus, which is located 195 bp away from SNP2 with high LD, are located in the genic region of GRMZM2G009320 (Chr7: from 164,954,304 to 164,956,841 kb). The region 4.5 kb upstream and downstream of SNP2 was scanned, since LD decay distance for chromosome 7 was 4.5 kb. There is only one other gene, GRMZM2G009538 (Chr7: from 164,948,659 to 164,953,684 kb), is located downstream of SNP2 (within 1 kb away; Supplementary Figure S1). Physical locations of GRMZM2G035688 and GRMZM2G009320 were updated to reference genome version 5 (Supplementary Figure S1).

## Discussion

These results demonstrated in maize for the first time that quantitative height loci first discovered through GWAS testcrossed diversity panel studies also conferred effects across four very diverse genetic backgrounds. An uncommonly discussed advantage of GWAS over linkage mapping is the ability to detect alleles that function nonspecifically across genetic backgrounds, maximizing discovery of context-independent alleles unaffected by genetic background epistasis that has hindered use of quantitative loci in the past. These alleles were first confirmed in linkage mapping populations (F_3:4_) developed from parental lines segregating for the two SNPs of interest (Chen 2016). However, Chen (2016) estimated different absolute effect sizes for these loci compared to those estimated in the initial GWAS (Farfan *et al.* 2015).

Across many studies, thousands of maize loci have been associated with agronomic traits in maize (*e.g.*, Thornsberry *et al.* 2001; Andersen *et al.* 2005; Weng *et al.* 2011; Larsson *et al.* 2013; Li *et al.* 2013; Peiffer *et al.* 2014; Farfan *et al.* 2015; Anderson *et al.* 2018). Although strong population structure and relatedness has been controlled in most GWASs to reduce false positive results (Lipka *et al.* 2015; Myles *et al.* 2009), we are cautioned by the cryptic population structure of *dwarf8* (Larsson *et al.* 2013) and possibilities of overfitting GWAS models to identify noncausal loci. Independent genetic confirmation of loci from GWASs is therefore necessary to understand whether the alleles are robust and useful as well as if the effect sizes are consistent across genetic backgrounds. Therefore, it is critically valuable that the two loci used in this study were validated over HIFs from four linkage populations, as contributing to taller PHTs in both ruler measurements and UAS data.

### Temporal resolutions of loci effects on PHT

The first seven UAS flights, flown during vegetative growth (typically up to 70 days after planting), found the largest effect sizes of loci and interaction effects of loci (Figures 3–6) as well as explained the most variation (Tables 1 and 2). This was unexpected since these SNPs were initially discovered in the GWAS panel through terminal height measurements using a ruler (Farfan *et al.* 2015). However, UAS phenotyping technologies were not available when Farfan *et al.* 2015 was conducted and temporal ruler measurements would have been infeasible. The last four UAS flights were flown in the reproductive stage (days 70–100 after sowing) after vegetative growth when internodes had stopped increasing and the effect size of loci and their interactions had become much smaller, in agreement with ruler measurement results taken July 2nd, 2019 (82nd day after sowing, between R5 and R6) (Tables 1 and 2; Supplementary Tables S4 and S5). In the reproductive growth phase, measuring plants individually with a ruler and plots by UAS, the differences between the main effects of loci could still be resolved (Supplementary Table S4 and S5). Maize yield has been most strongly correlated with PHT, in V6 (6-leaf), V10 (10-leaf), and V12 (12-leaf) growth stages, with V10 and V12 growing stages more important than other stages when earliness was desired (Yin *et al.* 2011). While no other studies have looked at maize yield relationships with height at intermediate growth time points, strong correlations have been reported between terminal PHT and grain yields in Texas maize (Anderson *et al.* 2019; Farfan *et al.* 2013). Context-dependency effects of loci under different genetic backgrounds were best able to be resolved in early UAS flights with larger effects sizes for populations 1 and 2 in the earliest flights (Figures 4 and 6). Population 3, developed as a reciprocal cross of population 2, was also observed to have had effect size differences (Figure 4).

### Pleiotropy of loci with flowering times

Both loci in this study were found to have pleiotropic effects on flowering (Supplementary Figure S5) not observed in the initial GWAS (Farfan *et al.* 2015). This was likely because heterosis in hybrid backgrounds tends to reduce or compress variation seen in inbred lines and because heterosis causes maize to flower earlier. Here the earliest flowering population had the smallest difference between alleles (population 1, <0.5 days) while the latest flowering population had and was able to discriminate the largest differences (population 3, >2 days) (Supplementary Figure S5).

### Description of candidate genes

GRMZM2G035688, within 5.8 kb of SNP1, corresponding to *aberrant phyllotaxy1* (also known as *abph1*) ,was first observed in maize mutant showing transformed phyllotaxy behavior (Jackson and Hake 1999). Phyllotaxy is the geometric arrangement of leaves and flowers to control the plant formation by shoot apical meristem (SAM). Unlike auxin action in phyllotaxy regulation in Arabidopsis (*Arabidopsis thaliana*), cytokinin-inducible type A response regulator is encoded by *abph1*, indicating that cytokinins play a role on aberrant phyllotaxy in maize (Lee *et al.* 2009). Auxin or its polar transport is necessity for *abph1* expression due to fact that *abph1* expression was dramatically lessened after treatment of a polar auxin transport inhibitor to maize shoots (Lee *et al.* 2009). Taken together, GRMZM2G035688 encoding *abph1* is essential for adequate maize *PINFORMED* (*PIN1)* expression, which is polar auxin transporter for leaf primordia expression in maize, and auxin localization in embryonic leaf primordia in SAM (Lee *et al.* 2009). Another gene, 1 kb away in the downstream region of SNP1, is GRMZM2G035637. This gene is the *Mo25* like gene that involves the cell proliferation, asymmetric cell establishment, as well as expansion that is crucial for plant establishment (Bizotto *et al.* 2018). This gene has not been previously implicated in PHT. However, given the pattern observed by UAV of stronger differentiation in alleles at early growth stages, when cells are dividing rather than expanding, this candidate is just as logical as *abph1*.

GRMZM2G009320, within 4.5 kb of SNP2, encodes a GAPDH, which catalyzes the sixth step of glycolysis into energy as well as carbons in higher plants. Under stress conditions such as salt or oxidative stresses, the activity of enzyme increases to manipulate energy formation in plants (Bustos *et al.* 2008; Zhang *et al.* 2011). Another gene 1 kb away in the downstream region of SNP2 is GRMZM2G009538. This gene is a member of the acidic leucine-rich nuclear phosphoprotein 32 (*Anp32*) family that involves in crucial biological process such as the regulation of cell signaling, transduction, and cell formation (Matilla and Radrizzani 2005).

### Recent breeding has selected the favorable alleles at both loci

**Figure 7:**
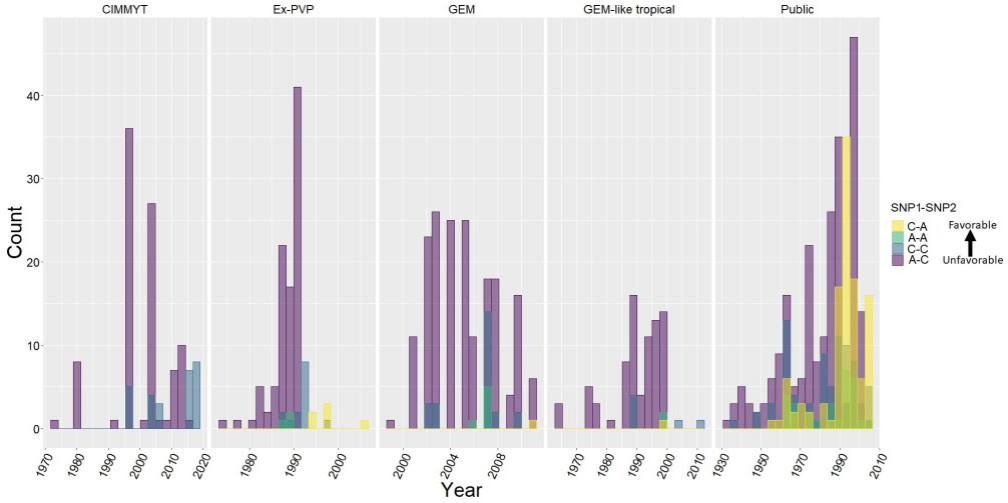
The allelic frequency combinations of SNP1 and SNP2 over years for five germplasm categories. The favorable C (SNP1) and A (SNP 2), referred to as XX, XX in this study, are both increasing in frequency in newer germplasm and are essentially fixed in US temperate Ex-PVP and public germplasm. The 989 subset of genotyped lines contained 448 public inbred lines, 87 GEM-like lines, 215 GEM lines, 118 Ex-PVP lines, and 121 CIMMYT germplasm lines.

Previously, several genes important in post domestication adaptation were identified by comparing maize lines from different early and late eras to show the proof of directional selection (van Heerwaarden *et al.* 2012); the genes of importance here (GRMZM2G035688 and GRMZM2G009320) were not included. Recent publicly available genotyping of diverse public inbred lines and germplasm (Romay *et al.* 2013; 989 subset containing 448 public inbred lines, 87 germplasm enhancement of maize (GEM)-like lines, 215 GEM lines, 118 Ex-PVP lines, 121 CIMMYT germplasm) for SNP1 and SNP2 information was extracted and grouped into five categories (Figure 7) and qualitatively compared by year of development or release. The frequency of SNP favorable alleles (X:X; increased height yield and flowering) showed consistent increases over time within most groups (Figure 7). Ex-PVP lines developed and released by industry and US public lines showed the greatest shifts toward the favorable alleles, almost to fixation. A lower frequency but less dramatic shift in CIMMYT originated tropical germplasm lines suggests that these loci still segregate in elite tropical maize, perhaps because the effects are less dramatic in the tropics. These alleles show favorable allelic selection over time, especially in temperate areas, unsurprising given their large phenotypic effects. This is another piece of evidence that these loci are economically valuable for improved varieties.

In summary, a previous GWAS field study of hybrids under stress successfully nominated quantitative trait variants (QTVs) that work across genetic backgrounds, in inbred lines and throughout diverse environments, confirmed through this study. New UAS tools provided substantially more information and better screening for the effects of these alleles than the traditional terminal ruler height measurements in which they were discovered. To get a better understanding of QTV’s affecting complex traits such as PHT and grain yield in maize, a combination of high-throughput phenotyping and genotyping studies must be evaluated together, which will be critical for managing the phenotypic plasticity of complex traits.
